# Absence of Viable *Toxoplasma gondii* in Artisanal Raw-Milk Ewe Cheese Derived from Naturally Infected Animals

**DOI:** 10.3390/microorganisms8010143

**Published:** 2020-01-20

**Authors:** David Ranucci, Elena Battisti, Fabrizia Veronesi, Manuela Diaferia, Giulia Morganti, Raffaella Branciari, Ezio Ferroglio, Andrea Valiani, Francesco Chiesa

**Affiliations:** 1 Department of Veterinary Medicine, University of Perugia, San Costanzo Street 4, 06126 Perugia, Italy; david.ranucci@unipg.it (D.R.); manuela.diaferia@unipg.it (M.D.); morganti.giulia@alice.it (G.M.); raffaella.branciari@unipg.it (R.B.); 2 Department of Veterinary Science, University of Turin, Paolo Braccini Square 2, 10095 Grugliasco (TO), Italy; elena.battisti@unito.it (E.B.); ezio.ferroglio@unito.it (E.F.); francesco.chiesa@unito.it (F.C.); 3 Istituto Zooprofilattico dell’Umbria e delle Marche “Togo Rosati”, Salvemini Street 1, 06126 Perugia, Italy; a.valiani@izsum.it

**Keywords:** *Toxoplasma gondii*, sheep, fresh cheese, LAMP, RT-PCR

## Abstract

The presence of viable *Toxoplasma gondii* was investigated in artisanal cheeses made from milk of naturally infected ewes. Ewe milk was analyzed beforehand for the presence and vitality of *T. gondii* by loop-mediated isothermal amplification (LAMP) and reverse-transcriptase PCR (RT-PCR), respectively. Cheeses were prepared from raw milk following a traditional cheesemaking process. The cheese obtained from *T. gondii*-positive milk was analyzed by LAMP to detect *Toxoplasma* DNA-positive samples. RT-PCR was then carried out to assess the viability of the parasites in *T. gondii*-positive milk samples and fresh cheese, after 5 and 15 days of ripening. Physical-chemical parameters of cheeses were also investigated. All cheese samples derived from *T. gondii*-positive milk were positive according to LAMP, at both 5 and 15 days of ripening, while none of the samples were positive according to RT-PCR. Thus, while the presence of the parasite was demonstrated by the detection of specific DNA, the absence of detectable *T. gondii* RNA supports the hypothesis that changes in the chemical and physical characteristics occurring during the cheesemaking process and ripening period, could be sufficient to inactivate viable *T. gondii* in milk, minimizing the risk of human infection through consumption of raw sheep milk cheese.

## 1. Introduction

Toxoplasmosis is a worldwide zoonotic disease with harmful effects on human health [1]. *Toxoplasma gondii* in humans can be transmitted vertically, by passing the tachyzoites to the fetus through the placenta, or horizontally, by ingestion of either sporozoites (from oocysts) or bradyzoites (from tissue cysts). Moreover, milk can be a source of *T. gondii* too during host infection, as tachyzoites can be released into milk that becomes a possible source of infection to the newborn by its ingestion in unpasteurized or raw form [2]. The detection of *T. gondii* in milk is reported in different animal species that produce it for human consumption or for cheesemaking [3,4], and related to the possible transmission of infection to humans [5,6]. Risk assessment studies are not always convergent on that topic as a non-significant influence of milk even on consumers belonging to risk categories have been reported [7]. Survival of the tachyzoites in refrigerated milk for up to 7 days is reported [8], but there is evidence indicating that viable tachyzoites excreted in milk are inactivated by the acidic gastric environment. However, tachyzoites can occasionally pass the gastric barrier and lead to infection of the host [9], and human toxoplasmosis outbreaks due to the consumption of raw milk of small ruminants, including sheep, have been reported [10], especially in rural areas. For this reason, the role of milk as a transmission route for *T. gondii* to humans is still under investigation [11].

Traditionally, sheep milk is rarely consumed directly [12], being used mainly for cheesemaking, often without pasteurization [13]. The presence of infective *T. gondii* has been reported in homemade cow milk cheeses obtained from milk experimentally infected with *T. gondii* cysts, for up to 10 days under refrigerated conditions [14], and in fresh goat cheese made by cold-enzyme treatment of unpasteurized milk spiked with tachyzoites [15]. No data are available on the fate of *T. gondii* tachyzoites in milk from naturally infected sheep or in fresh artisanal raw ewe milk cheeses, despite these kinds of products being sold on the market [16].

The aim of the study was to investigate the presence and viability of *T. gondii* in unpasteurized milk from naturally infected sheep and, subsequently, in artisanal fresh cheese (‘Primo Sale’) after 5 and 15 days of ripening (final products). The role of the microbiological, chemical, and physical changes that occurred during the cheesemaking and ripening was also correlated to the inactivation of *T. gondii*.

## 2. Materials and Methods

### 2.1. Milk Collection and Cheesemaking

Eight local sheep farms, tested *Toxoplasma*-positive by immunological assay from previous surveys [17], were enrolled in the study. Twenty liters of bulk milk were collected from each herd and immediately stored in refrigerated isothermal containers for transportation to the laboratory, where samples were stored at 4–6 °C for a maximum of 48 h. Milk samples from each herd were divided into two batches of 10 L each, separately processed for cheesemaking. For each batch, a 50 mL aliquot was removed for extraction of nucleic acids and subsequent molecular analysis. Eight ‘Primo Sale’ cheeses, weighing approximately 120 g each, were made from each batch, giving a total of 128 cheeses made according to traditional cheesemaking practices. In brief, milk was heated to 37 °C and starter culture was then added (*Lactobacillus lactis* subsp. *helveticus* and *Streptococcus thermophilus* – Fermenti Lattici, Laboratorio Prodor, Bobbio, PC, Italy). After 30 min of rest, liquid calf rennet was added (Caglio Liquido, Laboratorio Prodor, Bobbio, PC, Italy) and coagulation took place within 1 h. The curd was then cut into pieces (approximately 3 cm in size), transferred to cylindrical cheese molds (10 cm diameter and 3 cm height), and manually pressed to drain the whey. The product was stored at 30 °C for 1 h to facilitate proliferation of lactic acid bacteria (LAB) and then stored at 7 °C overnight. Marine salt was added manually to the surface of the cheeses before storage at 10 °C and 90% relative humidity in a ripening chamber. Samples of cheeses, made from the milk batches tested *Toxoplasma*-positive by loop-mediated isothermal amplification (LAMP) and reverse-transcriptase PCR (RT-PCR), were subjected to analytical determination and tested again for the presence and viability of *T. gondii* by LAMP and RT-PCR, respectively, at time T_5_ (5 days after cheesemaking) and T_15_ (15 days after cheesemaking).

### 2.2. pH, Water Activity and NaCl Measurement

The pH of cheeses made with milk tested positive for the presence of *T. gondii* nucleic acids was measured by inserting a pHmeter probe (Crison Instruments, Barcelona, Spain) into the inner part of each cheese sample; water activity (a_w_) was measured by a hygrometer (Series 3 TB, Decagon Devices Inc., Pullman, WA, USA); finally, the salt content was determined by the Volhard method 935.43 [18]. All analyses were performed in triplicate.

### 2.3. Purification of Total DNA and RNA from Milk and Cheese Samples

Samples of raw milk and cheeses were submitted to DNA and RNA extractions in triplicate. For extraction of nucleic acids from milk, 50 mL samples were first centrifuged at 1000× *g* for 15 min at 4 °C to separate the fat, as indicated by Mura and colleagues [19].

The pellet was resuspended in 200 μL of phosphate buffered saline (PBS, pH 7.2) with 1 mL of RNAlater (Qiagen, Hilden, Germany) to stop the transcription, and washed by two repeated pellettings and resuspensions with a solution of 200 µL of TE buffer (1 mM EDTA, 10 mM Tris–HCl (pH = 7.6)), 300 µL of 0.5 M EDTA (pH 8), and 600 µL of RLT buffer (Qiagen, Hilden, Germany).

DNA extraction from samples was performed using a GenElute Mammalian Genome DNA Miniprep kit (Sigma-Aldrich, St. Louis, MO, USA), while RNA was extracted using a RNeasy mini kit (Qiagen, Hilden, Germany).

Nucleic acid extraction from two different cheeses was performed in triplicate at each of the times considered and both DNA and RNA were obtained from the core part of each cheese. Twenty milliliters of RNAlater solution (Qiagen, Hilden, Germany) was added to 5 g of cheese, homogenized in a stomacher machine, and stored at −80 °C. At the moment of extraction, 1 mL of the suspension was added to 9 mL of trisodium citrate (2% w/w), and 1 mL of this solution was centrifuged at 6000× *g* for 5 min at 4 °C. The pellet was washed with a solution of 1 mL of TE buffer and 1 mL of 0.5 M EDTA (pH 8), resuspended in 1 mL of TRI reagent (Sigma-Aldrich, St. Louis, MO, USA) with 80 µL of proteinase K (20 mg/mL). After the addition of chloroform, DNA was extracted following the manufacturer’s instructions, while the RNA was recovered from the aqueous phase and processed using a RNeasy mini kit (Qiagen, Hilden, Germany).

The quality of the extracted DNA/RNA was evaluated with regard to purity and integrity by submerged gel electrophoresis followed by image analysis using a Bio-Rad ChemiDoc XRS + Molecular Imager (Bio-Rad Laboratories Inc., Hercules, CA, USA), and by the OD 260/280 nm ratio, using a NanoDrop 8000 Spectrophotometer (Thermo Fisher Scientific Inc., Miami, OK, USA).

### 2.4. Loop Mediated Isothermal Amplification (LAMP)

The DNA extracted from each raw milk sample and from cheese obtained from *Toxoplasma* DNA-positive milk was used as template for LAMP amplification targeting the SAG1 gene, according to the literature [20]. Sterile water and *T. gondii* genomic DNA extracted from parasite cultures and identified by species-specific PCR and sequencing were used in each set of reactions as negative and positive controls, respectively. Sensitivity and specificity of the LAMP assay were assessed [21].

### 2.5. Reverse Transcriptase (RT)-PCR

In order to obtain cDNA (mRNA), RT-PCR was carried out on RNA extracted from each raw milk sample and from cheese obtained from milk samples assessed as *Toxoplasma*-positive by LAMP. The samples were subjected to RT-PCR in order to assess the viability of *Toxoplasma* DNA using a QuantiTect reverse transcription kit (Qiagen, Hilden, Germany), following the manufacturer’s instructions. The mRNA was used as template for a conventional PCR targeting the SAG1 gene, as previously reported [22].

Briefly, the PCR mixture contained 2.5 µL of 10× PCR buffer, 2.5 UI of HotStarTaq DNA Polymerase (Qiagen, Milan, Italy), 0.5 μL of triphosphate nucleosides (dNTPs) mix (10 mM of each dNTP; Sigma-Aldrich, St. Louis, MO, USA), 8 pmol of each primer, and 4 µL of cDNA, giving a total volume of 25 µL. Thermal cycler conditions were 95 °C for 15 min, followed by 40 cycles at 95 °C for 1 min, 62 °C for 1 min, 72 °C for 1 min, and a final elongation step at 72 °C for 10 min. Negative and positive controls were included in each reaction. PCR products were visualized on a 2% agarose gel with a UV transilluminator (GelDoc 1000, Bio-Rad, Hercules, CA, USA).

The validation of the SAG1 RT-PCR protocol adopted was assessed according to previous reports [22]. Briefly, *T. gondii*-free milk and cheese homogenates were added with serial 10-fold dilutions of RH strain tachyzoites obtained from Vero cell cultures, ranging from 10^4^ to 10^-3^. These milk and cheese specimens were then processed similarly to the samples to evaluate the presence of PCR inhibitors and to assess the sensitivity of the protocol. In order to evaluate the specificity, RNA extraction from RH tachyzoites inactivated by heating (100 °C for 20 min) was used as template for SAG1 RT-PCR amplification.

## 3. Results and Discussion

The bulk tank milk from two herds, out of the eight enrolled for the study, was found to be *Toxoplasma*-positive by LAMP and RT-PCR analysis. Not all the herds considered in the trial result were positive according to the biomolecular assay even though they had previously been found positive by Immunofluorescence Antibody Test (IFAT) [17]. The presence of tachyzoites in bulk tank milk depends on several factors such as the infection phase and the immunity status of the animals sampled [23]. So, as not all serologically positive animals eliminate the parasite in milk, and not all the animals in the herds may be positive for *T. gondii* [17], the serological results obtained by IFAT may not be in accordance with the PCR results.

Only the 32 cheeses relative to the batches obtained from the bulk milk assessed as *Toxoplasma*-positive LAMP and RT-PCR were further tested for chemical-physical traits and the presence/viability of *T. gondii* during the cheesemaking process.

**Figure 1:**
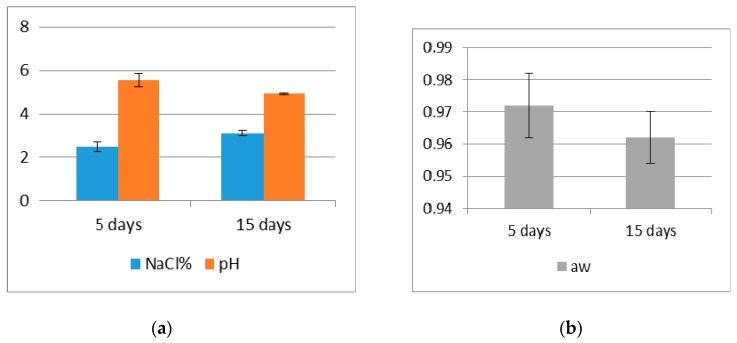
Chemical-physical results of raw-milk cheeses: (**a**) pH and NaCl content; (**b**) a_w_ values.

The results of the chemical and physical analyses and LAMP, and RT-PCR for *T. gondii* in milk and cheeses are reported in Figure 1 and Table 1, respectively.

**Figure 2:**
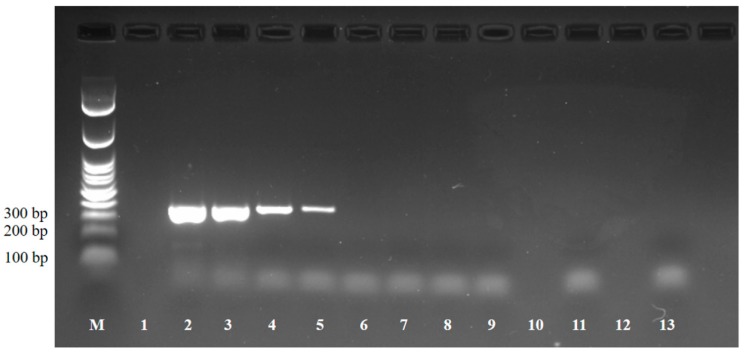
Sensitivity of SAG1 RT-PCR technique for detection of *T. gondii* tachyzoites RH strain. M marker in 100 bp ladder, Lane 1 empty space, Lane 2 RNA extracted by 10^4^ tachyzoites, Lane 3 RNA extracted by 10^3^ tachyzoites, Lane 4 RNA extracted by 10^2^ tachyzoites, Lane 5 RNA extracted by 10 tachyzoites, Lane 6 RNA extracted by 1 tachyzoite, Lane 7 RNA extracted by 10^−1^ tachyzoites, Lane 8 RNA extracted by 10^−2^ tachyzoites, Lane 9 RNA extracted by 10^−3^ tachyzoites, Lane 10 empty space, Lane 11 negative control, Lane 12 empty space, Lane 13 RNA extracted by tachyzoites inactivated by heating.

The pH dropped during ripening, due to LAB proliferation, notwithstanding a ripening temperature lower than that used for other processes for making cheese from ewe milk [24]. Whey draining and moisture reduction during ripening, due to the addition of NaCl, affected the a_w_ value. The salt content was in line with that reported in other raw-milk cheeses with a similar or even longer ripening process [13]. Thirty-two out of the 32 cheeses were found to be positive by LAMP both at T_5_ and T_15_. However, none of the cheeses tested positive by RT-PCR at either time considered. The sensitivity of SAG1 RT-PCR was found to be of 10 tachyzoites per reaction, and negative results were obtained from RH tachyzoites inactivated by heating (Figure 2).

In the present work, not only the presence of *T. gondii* DNA in raw milk was demonstrated, but also the viability of the parasites was considered by RT-PCR, to determine the possible role of ovine milk as a potential source of toxoplasmosis transmission. In fact, detection of the mere presence of *T. gondii* DNA in cheese, after different ripening periods, even through the use of specific amplicons (SAG1), is not evidence of its viability, and therefore does not provide valuable information on the risk to public health posed by raw-milk cheese in relation to toxoplasmosis.

RT-PCR was chosen as a screening method, as its use has been reported in *T. gondii* studies on tachyzoite–bradyzoite interconversion in humans where it provided a useful resource for monitoring stage conversion in vivo [25]. Moreover, quantitative RT-PCR has been proposed as a specific, sensitive, and rapid tool for estimating the viability of *T. gondii* oocysts [26]. RT-PCR detects mRNA, which is only produced by metabolically active parasites and could be a valuable strategy for the screening of viable tachyzoites in complex matrices to benchmark inactivation potential [27]. The in vivo mouse model is still considered the gold standard for *T. gondii* vitality and infectivity but is laborious and expensive [28] and there are ethical concerns associated with it [29]. The in vitro cell culture system, an alternative to mouse bioassay, must be optimized for assessment of vitality in complex food matrices, such as cheeses, and may require a long time to reduce assay risk [27,29]. Using RT-PCR, there could be a possible overestimation of the number of viable and potentially infective parasites [27] because mRNA persistence has been reported even in dead microorganisms [30]. However, we ruled out the presence of specific amplification by RT-PCR for RH tachyzoites inactivated by heating, thus confirming the absence of false positive results.

The absence of positivity by RT-PCR was highlighted as early as five days of ripening, before the more important changes in the chemical and physical characteristics of the cheese took place. As no heat treatment was performed during the cheesemaking, the effect on the viability of *T. gondii* could be due to the change in cheese pH (mean value of 5.5), essentially due to LAB growth, and the salt content (mean value of 2.48%) that are likely to represent unsuitable environmental conditions for the tachyzoites. Limited data on the survival of tachyzoites in cheesemaking technologies are available in the literature [15], but in dry fermented meat products, the addition of salt (> 1.3%) and the change in pH (final pH between 4.6 and 5.2) are able to inactivate even *T. gondii* tissue cysts in 4 h [31]. In the products analyzed, the pH was above the aforementioned range, but both the ripening period and NaCl content were higher. It is likely that the further process of ripening, with a drop in pH to more acidic levels and increase in NaCl concentration, may affect, to a stronger extent, the survival of the protozoan in artisanal raw-milk cheeses.

## 4. Conclusions

Raw-milk cheese made from naturally infected ewe milk is likely to bear *T. gondii* tachyzoites, as naturally infected sheep shed the parasite in their milk. Based on the results of this study, it can be concluded that cheesemaking procedures are able to inactivate the parasite, which was not found with RT-PCR after 5 days of ripening, in the absence of a pasteurization step. The study, however, did not provide quantitative data related to the amount of *T. gondii* in the sampled milk. In this respect, further studies are needed to define the fate of viable *T. gondii* in the case of high loads of tachyzoites in the milk, before cheesemaking.

## Figures and Tables

**Table 1:** Loop-mediated isothermal amplification (LAMP) and RT-PCR analysis of bulk milk and raw-milk cheeses obtained from ewe milk naturally infected with *Toxoplasma gondii*, at 5 and 15 days of ripening.

*Toxoplasma gondii*-Positive ^1^	Milk	Cheese	
		5 Days of Ripening	15 Days of Ripening
LAMP	16/16	32/32	32/32
RT-PCR	16/16	0/32	0/32

^1^ Number of positive samples/number of tested products.
